# Harnessing meta-analyses’ insights in ecology and evolution research

**DOI:** 10.1098/rsos.250759

**Published:** 2025-10-15

**Authors:** Pietro Pollo, April Robin Martinig, Ayumi Mizuno, Kyle Morrison, Patrice Pottier, Lorenzo Ricolfi, Jesse Tam, Coralie Williams, Yefeng Yang, Szymek Marian Drobniak, Malgorzata Lagisz, Shinichi Nakagawa

**Affiliations:** ^1^ Evolution & Ecology Research Centre, School of Biological, Earth & Environmental Sciences, University of New South Wales, Kensington, New South Wales, Australia; ^2^ School of Environmental and Life Sciences, University of Newcastle, Newcastle, New South Wales, Australia; ^3^ The Okanagan Institute for Biodiversity, Resilience, and Ecosystem Services, The University of British Columbia Okanagan, Kelowna, British Columbia, Canada; ^4^ Department of Biological Sciences, University of Alberta, Edmonton, Alberta, Canada; ^5^ Division of Ecology and Evolution, Research School of Biology, The Australian National University, Canberra, Australian Capital Territory, Australia; ^6^ School of Mathematics and Statistics, University of New South Wales, Sydney, New South Wales, Australia; ^7^ Institute of Environmental Sciences, Faculty of Biology, Jagiellonian University, Krakow, Poland

**Keywords:** impact factor, meta-regression, moderators, publication bias, scientific references

## Abstract

Meta-analyses are powerful tools to synthesize the literature in several fields of study, including ecology and evolution. However, it remains uncertain whether ecologists and evolutionary biologists fully comprehend meta-analyses’ findings or effectively apply them when citing these studies in their own research. Here, we first discuss key meta-analytical concepts and provide a guide to researchers in ecology and evolution on how to harness meta-analyses’ insights. For instance, we clarify the meaning of effect sizes and heterogeneity to improve understanding of meta-analyses’ quantitative findings. In addition, we analysed articles published in 2023 in ecology and evolution to investigate how frequently and in what context meta-analyses were cited. We found that approximately 21% of articles cited at least one meta-analysis, and that the relative number of citations of meta-analyses (0.62% of all citations analysed) was greater than the publication frequency of meta-analytical articles (0.44% of all articles). Most importantly, we found that while the direction of mean effect sizes from cited meta-analyses was often mentioned, the magnitude of effect sizes and the limitations of the data analysed were frequently overlooked. These findings underscore the need for improved citation practices of meta-analyses in ecological and evolutionary research, which our recommendations seek to promote.

## 1. Introduction

The research literature on ecology and evolution has rapidly grown in the past few decades [1,2]. Thus, it is no surprise that syntheses of the primary literature, such as systematic reviews and meta-analyses, have become common in this field [3]. While systematic reviews aim to summarize studies on a given subject, meta-analyses additionally test hypotheses by quantifying a mean effect size using data extracted from these studies [3–5]. Consequently, meta-analyses are often seen as powerful sources of robust summaries and predictions, commonly receiving more citations than empirical studies [6]. Despite their potential, insights provided by meta-analyses can remain obscure to readers who are less familiar with meta-analytical methods. If this is the case for many ecology and evolution researchers, citations of meta-analyses may be under-utilized or even misleading in this field of study.

In this article, we briefly explain concepts that are pivotal to meta-analyses (more in-depth discussions about them can be found elsewhere; e.g. [4,5,7–9]). We also provide a set of clear and concrete recommendations to effectively harness the information provided by these studies (§1.1). Afterwards, we explore how articles in ecology and evolution cite meta-analytical studies (§2). More specifically, we estimate how often meta-analyses are used as references (i.e. citation patterns) and explore the context in which these citations are harnessed in recent ecology and evolution research papers, analysing the specific content of meta-analytical citations from a representative sample of studies.

### 1.1. How to harness the insights of meta-analytical papers

#### 1.1.1. Search for meta-analyses

Meta-analyses have the advantage of representing ‘many articles in one’ as they compile and provide quantitative estimates of the existing literature on a particular topic [10]. This means that citing a meta-analysis represents strong support for an argument in addition to citing the most relevant empirical articles as examples. Thus, researchers should search for and cite meta-analyses that are relevant to the statements they make in their papers. The absence of a meta-analysis on a given topic can also be informative, although it requires a deeper understanding of the existing literature on that subject. This is because the lack of meta-analyses on a topic suggests either that the literature on that topic is scarce (which can be used to highlight the value of producing more empirical research) or that the literature on that topic would benefit from a synthesis (representing an opportunity for researchers interested in it to conduct a meta-analysis).

#### 1.1.2. Understand key concepts: effect sizes

Meta-analysts usually calculate effect sizes (i.e. standardized unitless estimates; e.g. Cohen’s *d*, Fisher’s *Zr*, odds ratio, response ratio [11]) from the data they collect (regarding a certain effect or relationship), allowing them to pool data across multiple studies. A mean effect size, obtained by fitting a meta-analytical model on individual effect sizes, represents an average effect or relationship across studies. Effect sizes convey not only the existence and direction of effects or relationships but also their magnitude [11,12]. Although many researchers focus exclusively on the direction of mean effect sizes (e.g. whether they are negative, positive or not different from zero), doing so can conceal critical information, thus representing a dangerous approach. For instance, textbooks often mention the positive relationship between bib size (patch of black plumage) and dominance status in male house sparrows as evidence that ornaments can serve as signals to conspecifics (e.g. [13,14]). Yet, a recent meta-analysis showed that this relationship is weak (*Zr* = 0.2), suggesting that this case is not a good example of the mentioned hypothesis [15] (see also [16]). This shows that qualitative information represents only part of the puzzle and can even be misleading in certain situations as, with a large enough sample size, even tiny effect sizes tend to statistically differ from zero [11]. Therefore, when mentioning the findings of a meta-analysis in a research paper, we recommend stating the magnitude of the mean effect size estimated in addition to its direction. This can be done by specifying the reported effect size value and its exact interpretation (e.g. log response ratio (lnRR) = 0.1, which translates to 10.5%). One can also use words that convey the magnitude of effect sizes (small or weak, medium or moderate, large or strong; table 1; electronic supplementary material, table S1). These terms represent interpretations based on benchmarks proposed by [12], but these can be arbitrary values only available for some effect size statistics (table 1). Moreover, when possible, we recommend avoiding vague terms such as ‘substantial’ and ‘significant’ as they do not properly convey information on the magnitude of effect sizes (electronic supplementary material, table S1).

**Table 1.** Benchmarks used to interpret the magnitude of various effect size statistics (based on [12] or conversions from it). Note that mentioning actual values (e.g. *r* = 0.2) is preferred over using the terms below.

effect size statistic	small/weak	medium/moderate	large/strong
Cohen’s *d*	0.2	0.5	0.8
Pearson’s *r*	0.1	0.3	0.5
Fisher’s *Zr*	0.1	0.31	0.55
log odds ratio (logOR)	0.36	0.91	1.45

#### 1.1.3. Understanding key concepts: heterogeneity

Meta-analyses quantify the variability among effect sizes that cannot be explained by chance alone (i.e. heterogeneity; e.g. *Q*, *I*^2^, *τ*^2^) [5,17]. For instance, total *I*^2^ quantifies the proportion of variation across effect sizes due to real differences rather than sampling error [17]. In ecology and evolution, heterogeneity across effect sizes is commonly high, likely because studies greatly vary, both methodologically and taxonomically [18]. High heterogeneity indicates that the mean effect size found lacks generalizability, i.e. the effect or relationship investigated is case-dependent [19]. For example, on average, the strength of male mate choice differs only slightly between males of varying competitive ability (e.g. size [20]). However, the variation across effect sizes is greater than what would be expected from random sampling alone, meaning that these differences are much larger in some cases (e.g. [21]). This highlights an important limitation: while mean effect sizes provide useful insights, they may not always reflect consistent patterns when variation across effect sizes is high. Fortunately, statistical methods allow us to break down this variation and identify its underlying sources [22]. For instance, sexual signal conspicuousness is moderately linked to attractiveness to mates, and while overall variation in effect sizes is high, differences between species contribute very little to this variation (i.e. *I*^2^_species_ < 5%), suggesting that this pattern may hold across taxa [23]. Still, high overall heterogeneity reveals that more studies need to be conducted if moderators and meta-regressions cannot explain much of the variation observed in the data (see below), which is often the case in ecology and evolution (see [24] for an exception).

#### 1.1.4. Understand key concepts: moderators and meta-regressions

Meta-analyses often investigate the effect of certain variables (i.e. moderators) on effect sizes, attempting to elucidate some of the variation in the data they collected and potentially providing further insights. In meta-analytical models, these variables are inserted as fixed factors, which can be categorical (i.e. different subsets of the data; e.g. vertebrates versus invertebrates) or continuous (the analysis then becomes a ‘meta-regression’). An example of the latter can be seen in a meta-analysis examining the effect of conspicuous patterns in lepidopterans (including the ones that resemble eyes) as an anti-predator mechanism, in which the size of the patterns was found to increase the deterrence of avian attacks in 11.6% for every (logarithm-transformed) mm^2^ beyond the average pattern size [25]. However, it is also important to consider the amount of variation explained by these variables (i.e. *R*^2^; see [26]). For instance, the aforementioned meta-regression using the size of conspicuous patterns in lepidopterans explained only 8.56% of the variation across effect sizes, indicating that other factors may play a role as well. Still, no or weak effects in meta-regressions can also provide powerful insights, as in a meta-analysis showing that air pollution hinders the performance of invertebrates regardless of the pollutant concentration [27]. In addition, accounting for some moderator variables may aid the interpretation of results by standardizing methodological differences (e.g. temperature or dosage differences) between studies (‘nuisance heterogeneity’ [28]).

#### 1.1.5. Understand key concepts: publication bias

By interpreting patterns in the data, meta-analyses can potentially identify when studies with non-significant results were left unpublished (i.e. publication bias [4,5,29]). This is important as meta-analytical results may be distorted when publication bias exists [30]. Even though there are methods to correct this (e.g. trim-and-fill and PET-PEESE; see [9,30,31]), finding signs of publication bias reveals a limitation of the study and a problem in the literature, indicating that further empirical studies are worthwhile.

#### 1.1.6. Identify knowledge gaps

Meta-analyses aim to be comprehensive and thus can reveal crucial knowledge gaps, such as methodological and taxonomic biases. For instance, research articles focusing on birds are much more frequent than those focusing on other taxa in several topics related to ecology and evolution (e.g. animal behaviour [32]; biodiversity [33]; parental care [34]), which consequently affects meta-analyses’ datasets [35]. Even though meta-analyses should clearly state such knowledge gaps, many simply omit this information [35]. Thus, researchers need to remain vigilant and be critical of the data presented in meta-analyses.

#### 1.1.7. Be critical

Researchers need to be as critical of meta-analytical articles as they are of other studies. More specifically, they must ascertain whether the meta-analyses they encounter transparently execute a plan that matches their questions and whether their limitations are fully disclosed (see sections above). By omitting information on their limitations, researchers often overestimate the importance and generality of their findings (see also [35]). Furthermore, the data, metadata and code underlying meta-analyses should be deposited in public repositories (e.g. OSF, Zenodo) to enable researchers to fully understand their methods and test their reproducibility and replicability (e.g. [16]). Note that PRISMA-EcoEvo guidelines advise meta-analysts on how to be transparent [36]. While we emphasize the need for meta-analyses to report key aspects, we acknowledge that subjective decisions are inevitably involved and that complete standardization may not be feasible. Although more meta-analyses are welcome in ecology and evolution, the overproliferation of non-transparent and low-quality meta-analyses may pose a concern to science [37], requiring researchers’ maximum care and attention to spot problematic meta-analytical articles.

#### 1.1.8. Use meta-analyses to justify your research and to discuss your findings

The above sections can hopefully improve the awareness of ecologists and evolutionary biologists, allowing them to ask fundamental questions about the meta-analyses they read (table 2). These questions should be especially vital when a meta-analysis encompasses the topic being investigated by the researcher, who should understand how their research project complements the literature and how similar their findings are to existing relevant data. For example, high heterogeneity, publication bias and knowledge gaps represent limitations of meta-analytic conclusions as well as of the existing literature. Knowledge gaps are particularly valuable to empiricists as further data collection that can help fill these gaps may represent a strong justification for research projects. On the other hand, mean effect sizes, moderators and meta-regressions should be compared and discussed in light of the new data collected. For instance, Postema [42] found that (artificial) larvae of the butterfly *Papilio troilus* suffered 7.1% fewer avian attacks when they had eyespots than when they did not have eyespots (but this only occurred when larvae were placed in rolled leaves). Postema’s [42] results could then be compared with the findings of a meta-analysis on the same topic across lepidopterans, which found a stronger effect of eyespots (compared with no eyespots) on avian predator avoidance (95% CI: 8.3–41.9% reduction, i.e. lnRR 95% CI = [0.08–0.35] [25]).

**Table 2.** Potential questions to harness the full potential of meta-analyses in ecology and evolution articles (cf. [8]) and to increase the value of empirical work in relation to previous related studies.

question	paper section	usage example
are there meta-analyses on a given topic?	§1.1.1	despite the existing wealth of data on the effect of heatwaves on plants, a meta-analysis on this topic is lacking
what is the direction and the magnitude of mean effect sizes reported?	§1.1.2	in amphibians, on average, parental care strongly increases egg survival (*Zr* = 0.54 [38])
how heterogeneous is the data (e.g. *I*^2^*, τ*^2^) presented, and what are their main sources?	§1.1.3	on average, male and female bird eggs barely differ in size (*Zr* 95% CI = −0.01 to 0.05), a result that is surprisingly consistent across effect sizes (*I*^2^_total_ 12.7% [24])
what predictor variables (moderators) were investigated and how much of the variation in the data (*R*^2^) can they explain?	§1.1.4	in mosquitofish, sex ratio explains some of the variation in the data regarding the effect of male size on reproductive performance (marginal *R*^2^ = 0.1 [39])
is there evidence of publication bias in the literature, and if so, how are mean effect sizes impacted by it?	§1.1.5	the conspicuousness of putative sexual signals weakly depends on individual condition (*r* = 0.17), yet evidence of publication bias in this literature reveals that this relationship is probably even weaker [23]
how representative is the dataset regarding, among other aspects, methodological and taxonomic coverage?	§1.1.6	a meta-analysis that claimed that ‘sex roles have been confirmed in nature’ [40] relied on data from only 66 species, of which just over a third were invertebrates (an incredibly diverse group that far outnumbers vertebrates in species richness)
how transparent and reliable is the meta-analysis?	§1.1.7	a meta-analysis examining the effect of temperature on sexual selection [41] included data that were not directly relevant to sexual selection, raising concerns regarding the reliability of its findings

## 2. Frequency and context of meta-analytical references in the literature

### 2.1. Material and methods

**Figure 1.**
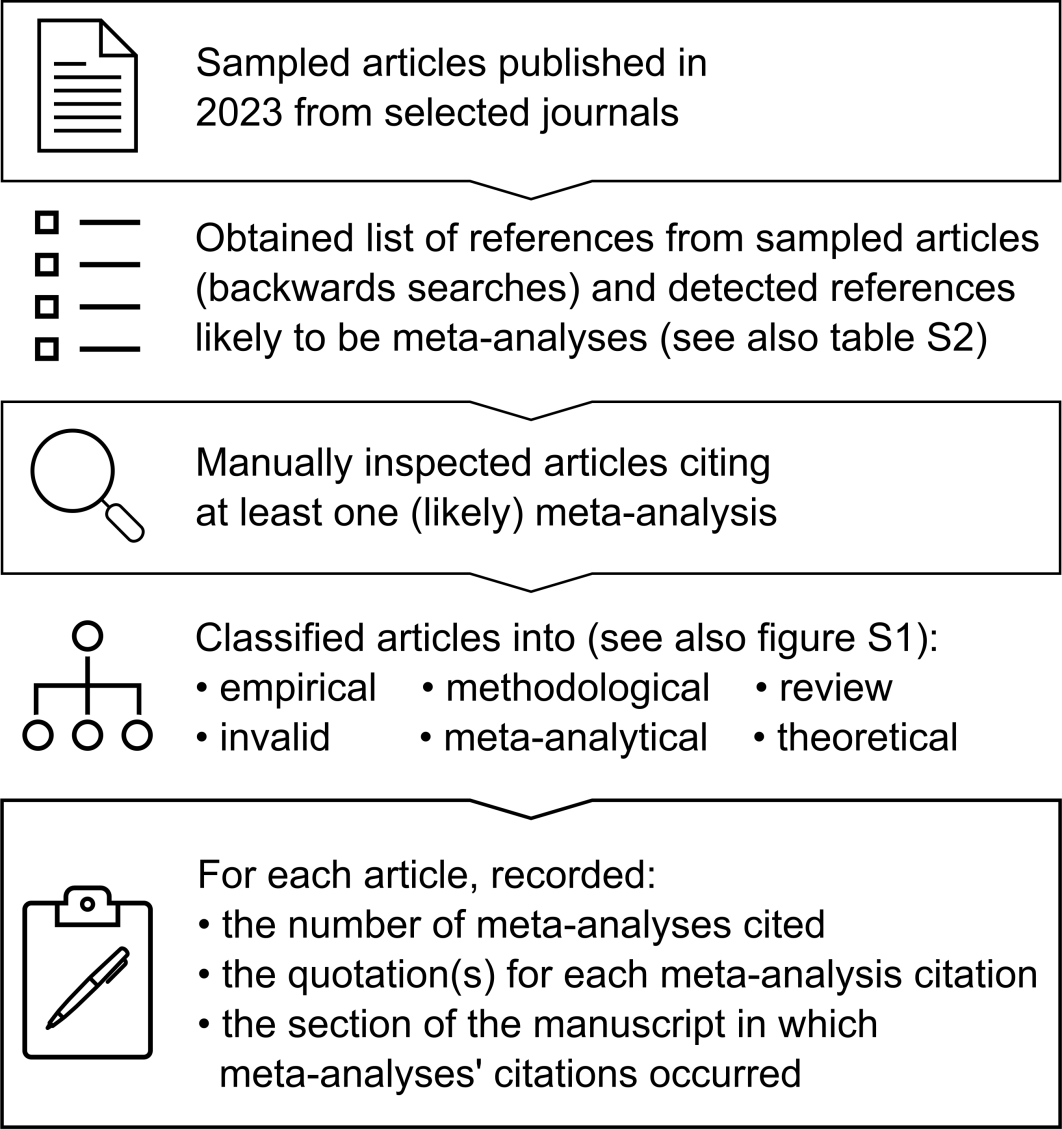
Methodology used in our study to examine the frequency and context in which meta-analyses are used as references by ecology and evolution research papers.

Our methodology, summarized in figure 1, was described in our pre-registration [43], and we adhered to it as much as possible (see deviations from the protocol described in electronic supplementary material, S1). We report author contributions using MeRIT guidelines [44] and the CRediT statement [45].

#### 2.1.1. Data collection

Pi.P. selected journals in ecology and evolution based on journal classifications by *Clarivate’s Journal Citation Records* (JCR) and *Scopus’ SCImago Journal Ranks* (SCR). More specifically, Pi.P. selected journals classified as ‘Ecology SCIE’, ‘Evolutionary Biology SCIE’, or ‘Behavioural Sciences SCIE’ by JCR, but only those that also had ‘Ecology, Evolution, Behaviour and Systematics’ as their first or second category according to SCR. This process resulted in the selection of 144 different journals. Pi.P. then obtained all articles published in 2023 by these selected journals using Scopus (accessed through the University of New South Wales), yielding a total of 17 145 articles. Using these articles’ DOIs, Pi.P. retrieved the reference list (i.e. backwards searches) of each article using the package *citationchaser* [46], which uses *the Lens* database, in R 4.4.0 [47]. However, no references were retrieved for 1393 articles due to absence of references reported by these articles or due to other reasons (e.g. errors from the database). For the remaining 15 752 articles, Pi.P. then verified, in an automated fashion (i.e. using references obtained from *the Lens* database for each article), whether the title of each reference cited by selected studies contained specific terms related to meta-analyses to ascertain which references were likely to be meta-analyses (hereby *meta-analytical references*; see electronic supplementary material, table S2). Pi.P. then selected 686 articles (from 120 journals) by randomly picking articles from as many journals as possible with at least one automatically detected meta-analytical reference. Afterwards, all authors inspected the full text of these 686 articles, classifying the type of the study (see electronic supplementary material, figure S1) and recording: (i) the number of meta-analytical references (manually assessed their titles for the terms listed in electronic supplementary material, table S2), (ii) the sentence in which these references were mentioned in the main text of the study paper (i.e. quotations), and (iii) the paper section where these citations appeared (i.e. ‘introduction’, ‘methods’, ‘results/discussion/conclusion’ or ‘other’). During these full-text paper inspections, we separated meta-analytical references, based on their title, into ‘true’ meta-analytical references (i.e. quantitative syntheses of the literature) and methodological meta-analytical references (i.e. papers on how to conduct meta-analyses).

We curtailed our sample size for most of the analyses (see below) from 686 to 645 articles because 41 articles were considered invalid (e.g. editorials, response letters) or did not cite any true meta-analytical references. We noticed that reference lists obtained from *the Lens* database tended to miss some references (i.e. underestimate the number of references cited), so we also obtained the number of references cited by the 645 inspected articles from *Web of Science*, which provided a more robust reference count. Furthermore, when exploring the occurrence of true meta-analytical references in articles, we only considered sections within the IMRaD structure (i.e. introduction, methods, results, discussion or clear amalgamations of these sections). For analyses, we grouped the paper sections ‘results’, ‘discussion’ and ‘conclusion’ together because some journals present these sections as one.

All authors (excluding S.M.D.) analysed quotations containing citations of true meta-analytical references, extracted from inspected articles (see above). We evaluated whether the quotation contained the following information related to the meta-analysis being cited: (i) results (of any kind), (ii) quantitative results (magnitude or variability of findings; electronic supplementary material, table S1), and (iii) limitations (e.g. gaps in the literature; electronic supplementary material, table S3). However, we highlight that these evaluations can be highly subjective and thus should be considered with caution.

#### 2.1.2. Statistical analyses

Pi.P. primarily reported descriptive results of the collected data. Unless stated otherwise, estimates reported in the paper represent mean ± s.e. Pi.P. also fitted generalized linear mixed models to examine associations between certain variables and three outcomes: (i) the number of true meta-analytical references, (ii) the total number of references, and (iii) the proportion of true meta-analytical references among all references. Pi.P. used a negative binomial error distribution for models with the first two outcomes as response variables, but used a binomial error distribution for models with the third outcome. For the first set of models, Pi.P. included article type as a predictor variable and journal name as a random effect. For the second set of models, Pi.P. included Clarivate’s 2022 impact factor of the journal in which articles were published as the predictor variable (standardized to zero mean and divided by s.d.) with journal name and article type as random factors.

Pi.P. performed all analyses in the software R v. 4.4.0 [47]. Pi.P. fitted generalized linear mixed models using the functions *glmer* and *glmer.nb* from the package *lme4* v. 1.1.35.5 [48]. Pi.P. performed pairwise comparisons (z-tests) using the function *glht* from the package *multcomp* v. 1.4.26 [49] and the function *cld* from the package *multcompView* v. 0.1.10 [50]. The code and data for our analyses are available at https://pietropollo.github.io/meta_impact/ and https://zenodo.org/records/16888338.

### 2.2. Results

#### 2.2.1. Frequency of meta-analytical references

We found that 21.2% of articles (3338 out of 15 752) whose references we evaluated with an automated approach cited at least one meta-analytical reference (i.e. reference title contained any of the terms from electronic supplementary material, table S2). Because many articles had no meta-analytical references, the average proportion of meta-analytical references per article from the total number of references cited was 0.62 ± 0.01%. However, we highlight that *the Lens* database failed to retrieve some references and added others, so this estimate may be imprecise. To put this estimate into perspective, we calculated the proportion of articles in ecology and evolution published in 2023 that were meta-analyses. Using the same detection method as the rest of our results (i.e. searching titles for terms in electronic supplementary material, table S2), we found that 0.44% of articles (70 out of 15 752) were meta-analyses. This means that articles in our dataset cited meta-analyses at a 40% higher frequency relative to the publication pattern observed in 2023.

**Figure 2.**
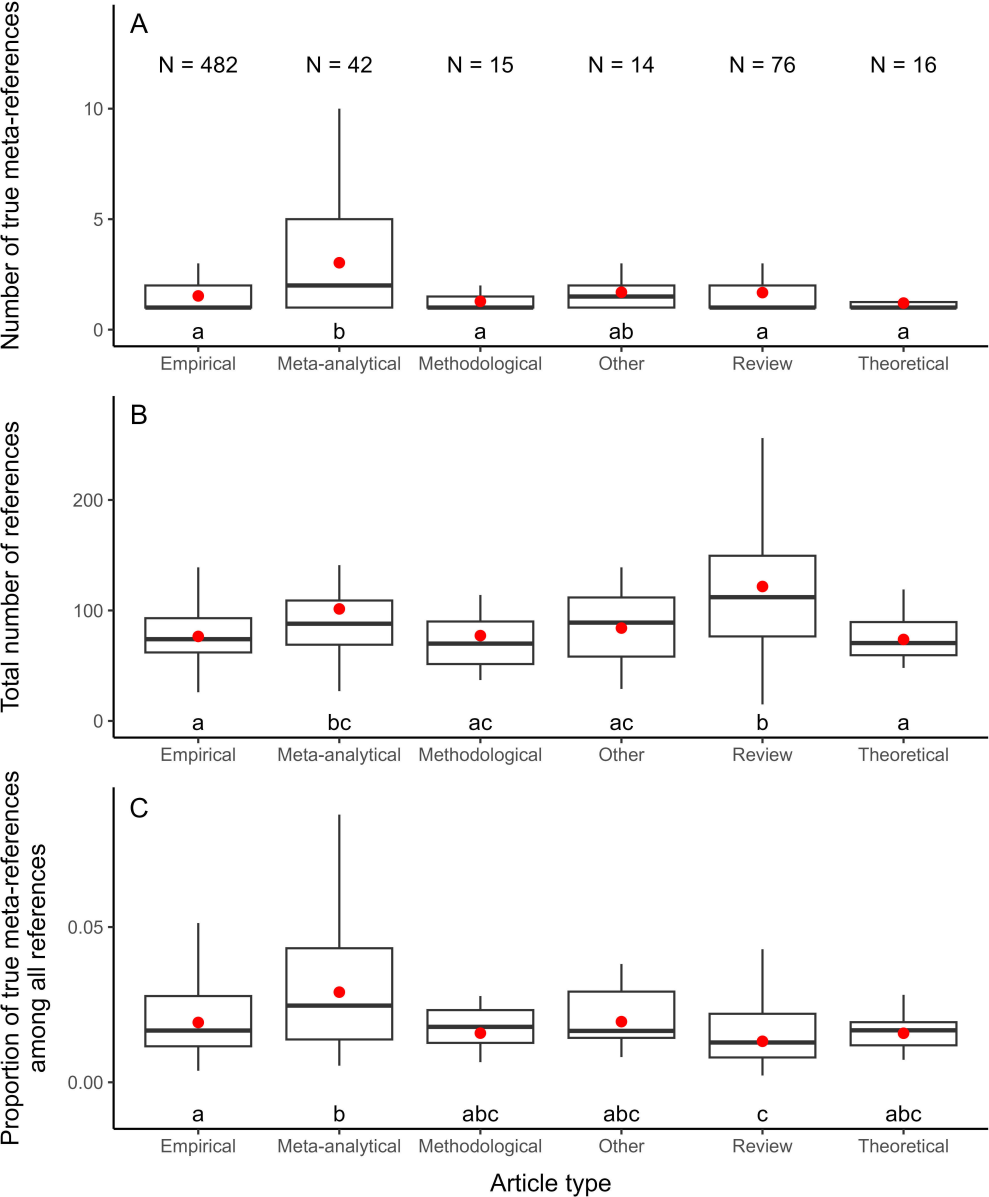
Number of true meta-analytical references (A), total number of references (B), or proportion of true meta-analytical references among all references (C) per article depending on article type. All articles shown cited at least one true meta-analytical reference. The boxes enclose 50% of the data (interquartile range), the whiskers contain values up to 1.5 times the interquartile range, and the solid line within the boxes represents the median. Outliers (values outside of 1.5 times the interquartile range) are not shown. Red dots represent the mean controlled by journal identity (see §2.1). Distinct letters represent statistical differences between article types (*z*-values with *p* < 0.05 for all pairwise comparisons).

Out of the 686 articles we manually inspected, 670 contained at least one meta-analytical reference. However, only 80.7% of manually detected meta-analytical references were true meta-analyses, while the remaining 19.3% of references were methodological papers (about meta-analytical tools or practices; e.g. [51]). Among the 645 articles that cited at least one true meta-analytical reference, the average number of meta-analytical references cited per article was 1.7 ± 0.05. We observed that true meta-analytical references were cited more often by meta-analytical articles than by other types of articles (except for articles whose type was classified as ‘other’; figure 2A). However, articles also varied in their total number of references: review articles and meta-analytical articles cited, on average, more references than most of the other article types (figure 2B). Considering this, on average, meta-analytical articles cited a greater proportion of true meta-analytical references (from the total number of references cited) than empirical articles (2.9% versus 1.9%, respectively; *z* = 4.08, *p* < 0.001), yet articles of both of these types contained proportionally more true meta-analytical references than review articles (1.3%; *z_meta-analytical vs. review_* = 6.14, *p* < 0.001; *z_empirical vs. review_* = 3.81, *p* = 0.002; figure 2C). By contrast, this estimate was similar between other types of articles (i.e. methodological, theoretical, or others; figure 2C). Furthermore, the proportion of true meta-analytical references was not associated with the impact factor of the journal in which articles were published (electronic supplementary material, figure S2).

#### 2.2.2. Context of meta-analytical citations

**Figure 3.**
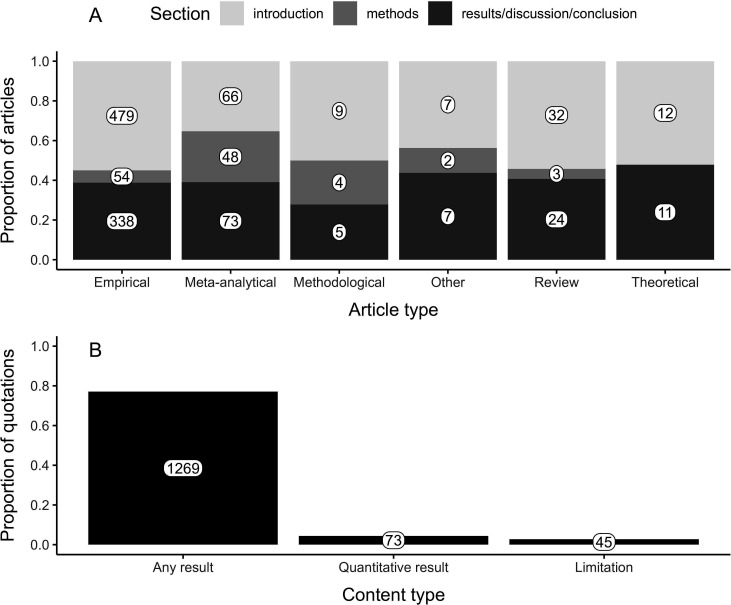
Proportion of (A) article sections in which true meta-analytical references were cited by article type and (B) the type of content of the sentence that contained true meta-analytical references (i.e. context of the citation).

Overall, 51.5% of true meta-analytical references were cited in the introduction section, 9.5% in the methods section, and 39% in the results, discussion, or conclusion sections. However, these proportions varied across distinct types of articles (figure 3A). For instance, true meta-analytical references were cited more often in the methods section in meta-analytical articles (25.7% of their meta-analytical references) than in empirical (6.2%, *z* = 7.56, *p* < 0.001) or review articles (5.1%, *z* = 3.03, *p* = 0.02), while this frequency among other article types was statistically uniform (figure 3A). Conversely, true meta-analytical references were cited more often in the introduction section in empirical articles than in meta-analytical articles (55% versus 35.3% of their meta-analytical references, respectively; *z* = 4.82, *p* < 0.001), with no differences among other article types regarding this frequency (figure 3A). By contrast, the frequency of citations in the discussion was uniform across article types (figure 3A).

Regarding the content of quotations associated with true meta-analytical references, 77.1% of them appeared to mention a result of the meta-analytical reference being cited (figure 3B). Nonetheless, a quantitative result was reported in only 5.7% of all quotations (figure 3B). Moreover, a limitation of the meta-analytical reference being cited (or of the literature it explored) was mentioned in 2.7% of all quotations we evaluated (figure 3B).

### 2.3. Discussion

We explored patterns related to citations of meta-analyses (i.e. meta-analytical references) in ecology and evolution articles published in 2023. We found that the proportion of meta-analytical references in these articles was greater than the frequency of publication of meta-analyses observed in the same year. We also found that the proportion of meta-analytical references relative to all references was greater in meta-analytical and empirical articles than in review articles (figure 2) and that this proportion was not related to the impact factor of the journal in which articles were published (electronic supplementary material, figure S2). Moreover, the location of meta-analytical references in papers varied across article types (figure 3A). Most importantly, we noticed that authors mainly mentioned the results of meta-analyses they cited yet rarely specified quantitative information or limitations from these meta-analytical references (figure 3B). Below, we discuss these findings in detail and reiterate some strategies to effectively harness insights reported by meta-analyses for researchers in ecology and evolution (but see §1.1).

Our finding that meta-analytical references in the literature of ecology and evolution occur at a higher rate than the publication of meta-analyses in this field for the period evaluated (2023) further supports that meta-analyses receive more citations than other article types [6]. Nonetheless, it is possible that many ecologists and evolutionary biologists lack the necessary knowledge about meta-analyses or simply do not have the habit of using meta-analyses to substantiate their claims. While some researchers may prefer citing articles other than meta-analyses, we hope that our study (especially §1.1) may convince and/or equip researchers to cite meta-analyses in their work when it is relevant to do so. We also note that, while we found that the proportion of meta-analytical references from all references may depend on the type of article published (figure 2), other factors could also influence citation patterns of meta-analyses. For instance, the frequency of meta-analyses and meta-analytical references could vary across subdisciplines of ecology and evolution, investigated taxa, or journal characteristics beyond impact factor, so we encourage further assessments of the literature to reveal whether this is the case.

We found that researchers mainly mention the results of meta-analyses they cited, which is congruent with the fact that most meta-analytical references are in sections meant to present the context of their study (e.g. introduction and discussion; figure 3A). However, we noticed that meta-analytical results mentioned in articles were qualitative in approximately 94% of the cases (figure 3B). This means that authors often omitted nuances about the magnitude of mean effect sizes or other quantitative results despite their importance to the interpretation of meta-analyses’ findings. Although meta-analyses may be at fault for this pattern if they do not properly convey the meaning of the mean effect size they report, reporting guidelines (e.g. PRISMA-EcoEvo [36]; ROSES [52]) attempt to minimize these cases as they recommend discussing meta-analytic results in light of the magnitude of mean effect sizes estimated (item 25.1 [36]). Perhaps researchers prefer reporting qualitative over quantitative information because they are unfamiliar with the concept of effect sizes (§1.1.2). This may stem from the fact that researchers in ecology and evolution often rely on null hypothesis significance testing to make inferences about their own findings [11]. However, this approach poses several problems, such as ignoring the magnitude of biological effects or relationships being investigated in research articles [11]. Therefore, we emphasize that researchers should adopt effect sizes in their research regardless of the approach used (i.e. not only in meta-analyses) to report their own results and to discuss findings of other studies (which has been continuously proposed for several decades [11,53–56]). This would raise awareness on how to interpret meta-analyses’ results as well as improve the communication of results from all types of research in the field of ecology and evolution.

Another pivotal issue we observed is that less than 5% of articles on ecology and evolution use meta-analytical references to highlight limitations of the existing data on a given topic (figure 3B; e.g. high heterogeneity, publication bias, knowledge gaps; see §1.1). We are concerned that researchers may view meta-analyses as the definitive conclusion or ‘final word’ when, in reality, they serve to highlight the current state of the field alongside existing gaps in knowledge. This might be a symptom of the publication system’s relentless pursuit of novelty, even though this represents an esoteric concept used by some to pretend they can predict the future impact of research projects [57–59]. Instead of treating meta-analyses as definitive answers to research questions, we argue that researchers should consider limitations in the data reported by meta-analyses to plan and justify their studies. In fact, researchers constantly find exceptions to the rule [60], which reveal that most norms are just perceived patterns from fragments of reality and thus ill-defined (e.g. the idea of sex roles [61,62]). We also appeal to those who act as gatekeepers (e.g. editors) to accept that further data collection is always valuable, so prioritizing high-quality research (e.g. well-designed, transparent) over perceived novelty represents an essential endeavour for scientific advancement.

Note that changes in research practices can substantially change how we assess the quality of meta-analyses. For example, how heterogeneity is measured and reported has dramatically changed (and continues to change) in recent years (e.g. [22]). Likewise, discussions on research transparency and data availability have only recently gained traction (e.g. [63,64]). Furthermore, the use of AI can influence how we monitor and use modern systems to curate current knowledge bases. For instance, tools to automate literature screening and data extraction have already been developed and, although they might not be good enough to replace human labour (e.g. [65,66]), they can potentially change how meta-analyses are conducted. Our recommendations for meta-analyses’ readers should remain valid despite these changes but may need future adjustments to reflect gold-standard practices and technological advancements related to meta-analyses.

Many of our results relied on a simple method to ascertain whether references were meta-analyses, i.e. searching for certain terms in reference titles (electronic supplementary material, table S2). However, titles of some meta-analyses do not contain such terms, which represents an important limitation of our results regarding the frequency of meta-analytical references in the literature. In our dataset, we classified 47 out of the 686 articles we manually inspected as meta-analytical articles and, from those, only 25 had titles with sought terms (i.e. sensitivity: 46.8%). For comparison, recent systematic maps of meta-analyses indicate that the proportion of meta-analytical articles containing at least one of the sought terms in their title varies: 46.1% in [35], 57.1% in [67] and 75% in [68]. This suggests that the number of meta-analytical references we observed in our dataset is likely to be underestimated. However, this should not affect the comparison we made between the observed and the expected proportion of meta-analytical references from all references cited because both use the same detection method, thus being comparable. Moreover, citation patterns may differ between meta-analyses with and without sought terms in their title if the former are more likely to be cited (exactly because their titles clearly denote they are meta-analyses). Furthermore, although our results related to how meta-analytical references were used in articles (e.g. paper section, content of quotations) could change by including these other meta-analytical references, this is unlikely as the content of meta-analyses should not depend on their title.

## 3. Conclusions

We provided a brief guide to ecology and evolutionary biologists on meta-analytical methods. More importantly, we included several suggestions on how researchers can fully harness the potential of meta-analyses they encounter, especially the ones overlapping with their research. Our suggestions are likely to remain valuable for researchers, regardless of advances in technological tools (e.g. AI) that may change how meta-analyses are conducted. Our recommendations are critical in light of the suboptimal use of meta-analytical references found in ecology and evolution research articles, including an overreliance on qualitative rather than quantitative meta-analytic findings and a lack of engagement with the limitations highlighted in meta-analyses. We thus hope that our guidance can improve this scenario by helping researchers to better incorporate meta-analytical findings in their own research.

## Data Availability

All data and code used in this study are available at https://pietropollo.github.io/meta_impact/, Zenodo [69], and OSF [70]. Electronic supplementary material is available online [71].
